# Interaction between Soil Moisture and Air Temperature in the Mississippi River Basin

**DOI:** 10.4236/jwarp.2017.910073

**Published:** 2017

**Authors:** Chunling Tang, Dong Chen

**Affiliations:** 1 Comutational Exposure Division, Environmental Protection Agency, Research Triangle Park, Durham, NC, USA; 2 Key Laboratory of Water Cycle and Related Land Surface Processes, Institute of Geographic Sciences and Natural Resources Research, Chinese Academy of Sciences, Beijing, China

**Keywords:** Soil Moisture, Air Temperature, Quantile Regression Model, Wavelet Transform Coherency, Climate Change

## Abstract

Increasing air temperatures are expected to continue in the future. The relation between soil moisture and near surface air temperature is significant for climate change and climate extremes. Evaluation of the relations between soil moisture and temperature was performed by developing a quantile regression model, a wavelet coherency model, and a Mann-Kendall correlation model from 1950 to 2010 in the Mississippi River Basin. The results indicate that first, anomaly air temperature is negatively correlated to anomaly soil moisture in the upper and lower basin, and however, the correlation between them are mixed in the middle basin. The correlation is stronger at the higher quantile (90^th^) of the two variables. Second, anomaly soil moisture and air temperature show strong coherency in annual frequency, indicating that the two variables are interannually correlated. Third, annual air temperature is significant negatively related to soil moisture, indicating that dry (wet) soil leads to warm (cool) weather in the basin. These results have potential application to future climate change research and water resource management. Also, the strong relationship between soil moisture and air temperature at annual scale could result in improved temperature predictability.

## 1. Introduction

Increase in temperature has been observed as a consequence of climate change over the last century. Air temperature (T) is anticipated to continue increasing in the future, while the mechanisms for the increase remain uncertain [1] [2]. Soil moisture (SM) has been shown to have influence on T by controlling the partitioning between sensible and latent heat, and interaction between SM and T accounts for about two-thirds of the climate change signal in the future climate models [2]. A better understanding of the relationship between them might provide sharper insight into these mechanisms [3] [4] [5] [6]. With this concept in mind, in this study, we investigated the relation between SM and T and the results are essential for understanding the global temperature rises specifically and global warming more generally.

Several studies have pointed to the relation between SM and T to help explain projected global warming [3] [5] [7]-[12] and extreme climate events (e.g. drought and flood). Many studies show that interaction between SM and T plays a significant role in extreme climate condition by land atmosphere interactions [6] [8] [13] [14]. Two sets of apparently contradictory results have been presented, namely that a reduction in SM leads to an increased T [3] [9] [10] [11] [12] [15] [16], and while other studies indicate that a SM deficit leads to cold weather [3] [12] [16] [17] [18]. For example, positive feedbacks exist over some of Northeast Asia, even at a less significant level [3] [18] [19]. However, significant negative feedbacks between SM and T exist at wet transition zones in Northern China. SM deficit induces higher air T in the plains, well reduces the T over mountains in France from 1989 to 2008 in [20].

However, due to a lack of sufficient long-term and high-resolution SM data, systematically synthesizing the relationship between SM and T remains a significant challenge. Here we propose the first exclusive analysis between SM and T, to study the relations by a number of factors, including Quantile Regression Model (QRM), Wavelet Transform Coherency (WTC) analysis, and Mann-Kendall trends in the Mississippi River Basin (MRB). Many studies [10] [17] [21] showed that SM is a drought indicator, so, the interaction between SM and T helps both understanding the climate change and the climate extremes. The MRB, as the largest basin of the United States, spans a wide range of climatic regions with snow-dominated, transitional, and rain-dominated regions, the results of the research might provide an indication of the relation between SM and T for the whole nation.

## 2. Study Area and Model

### 2.1. Study Area

The study area is the MRB, which drains 41 percent (41%) of the water from all or part of the 31 states in the United States, and are home to over 100 globally important aquatic species such as walleye, sauger and perch. Furthermore, as the largest basin in nation, the MRB spans a wide range of climatic regions with snow-dominated, transitional, and rain-dominated regions. Climate changes have occurred and are anticipated to continue in the future. Many changes indicate this region is also undergoing a system wide response to climate change. Given the importance of the MRB in terms of environmental processes, nature resources, and economics, this study provided significant meanings for the MRB.

### 2.2. Variable Infiltration Capacity (VIC) Model

In MRB, long-term observation SM data cannot be obtained, therefore, the VIC [22] model was utilized to simulate SM for a daily time step at 1/8° spatial (about 12 km) resolution for the period of 1950 to 2010. The methods used to calibration of the simulation results are reported in more detail in [10] [23].

The VIC model is a macroscale, grid-based water and energy balance model, which has been successfully applied to many large river basins with good results [22]-[28]. Distinguishing features of the VIC model include the sub-grid variability in SM, land surface vegetation, precipitation, and topography in use of the elevation bands.

In this study, the VIC model is forced with parameter files and meteorological forcing. The parameter files include soil parameters, vegetation parameters, and elevation bands. The soil parameter files describe the unique soil properties for each grid cell, the main parameters include soil texture information, soil bulk densities, hydraulic conductivities, and thickness of each soil layer, infiltration capacities, and base flow parameters. The VIC model uses a complex and explicit conceptual framework in accounting for variations in SM. The soil was divided to three layers with depth of each layer specified for each grid cell as derived during model calibration in this study. The thin upper layer (~10 cm) simulates runoff processes in near surface soil layers, and thicker second layer (50 ~ 80 cm) and third layer (100 cm ~ 250 cm) represent slower storage and drainage processes in this study. Deep layer SM has a longer memory than many other land surface variables (such as precipitation and temperature) because SM reservoir is a larger storage term (than evapotranspiration and snowpack) in the water balance [21]. SM of the third layer was used in details for the analysis because of its consistency and longer memory in the study. The daily simulated SM are aggregated to month SM then converted to anomalies by subtracting the mean monthly SM for each model grid cell from 1950 to 2010.

The vegetation parameters describe different land cover characterization, which has a total of 14 different land cover classes. The primary characteristic of the land cover that affects the model simulation is Leaf Area Index (LAI). The elevation band files define the properties of each elevation band to improve the performance of the model in changing topography, especially mountainous regions (upper MRB). Four elevation bands were used in this study.

The meteorological data include observed precipitation, maximum temperature, minimum temperature, and wind. Daily precipitation and temperature were obtained by interpolating daily observation data from National Weather Service Cooperative Observe with scaling for topographic effects using the Parameter-elevation Regressions on Independent Slope Model (PRISM) precipitation map [23]. Daily wind speed was obtained from National Centers for Environmental Prediction (NCEP) [23].

### 2.3. Model Calibration

The VIC model was calibrated at six United States Geological Survey (USGS) gaging stations for which stream flow observations were available from 1950 to 2010.

The VIC model calibration includes calibrating six parameters: a) the infiltration parameter which controls the partitioning of rainfall (or snowmelt) into infiltration and direct runoff; b) the second and third soil layer thicknesses, which affect the water available for transpiration and baseflow respectively; c) *Ds*_max_, *Ds*, and *Ws*, which are baseflow parameters and also are estimated. *Ds*_max_ is the maximum baseflow velocity, Ds is the fraction of maximum baseflow velocity, and *Ws* is the fraction of maximum soil moisture content of the third soil layer at which non-linear baseflow occurs. These three baseflow parameters determine how quickly the water stored in the third soil layer is evacuated as baseflow [22].

A 30-year period (1950–1979) of the 60-year record was utilized for the calibration process. This 30-year period encompassed a range of extreme climate conditions (e.g., flood and drought), and normal years for testing the two models’ performance. A separate 30-year period (1980–2009) was used for model validation. That period also encompassed a range of climatic conditions under which we evaluated the models’ performance. Two standard statistical techniques, the root meansquare error (RMSE) and the widely used coefficient of determination (*R*^2^) value, were used to test and evaluate the accuracy of the model simulations.

## 3. Methods

### 3.1. Quantile Regression Model (QRM)

QRM is a statistical technique intended to estimate, and conduct inference about conditional quantile functions. QRM is capable of providing a more complete statistical analysis of the stochastic relationships among random variables, which may best represent the true relationship between SM and air T.

The QRM can be expressed as:

$$y_{i}=\beta_{0}^{\left(p\right)}+\beta_{1}^{\left(p\right)}x_{i}+\varepsilon_{i}^{\left(p\right)}$$

where, 0 < *p* < 1 indicates the proportion of the population having scores below the quantile at *p*. where: *x*_*i*_ and *y*_*i*_ are pairs of the data, for based on the sample of the SM and air T. $\varepsilon_{i}^{\left(p\right)}$ is the error term, which is independent and identically distributed for *i* = 1, 2, ···, *n*. $\beta_{0}^{\left(p\right)}$ is the quantile-specific parameter.

QRM estimates multiple rates of change from the minimum to maximum and therefore can provide a more complete picture of the relationship between SM and air T. Previous studies have shown that SM is most strongly related to maximum temperature [5] [7]. In this study, the 5^th^, 10^th^, 25^th^, 50^th^, 75^th^, 90^th^ and 95^th^ quantiles with the 50^th^ quantile representing the median response and 5^th^ and 95^th^ representing the extreme climate conditions were used.

### 3.2. Wavelet Transform Coherency (WTC)

To furtherer understand the correlation between SM and air T; the WTC [29] [30] [31] was utilized. The continues wavelet transforms a one-dimensional time series to a two-dimensional time-frequency image simultaneously which shows both the amplitude of any periodic signals within the series and how this amplitude varies with time. The continuous wavelets transform *W*_*n*_ of a discrete sequence of observations *x*_*n*_ is described as following:

(1) $$w_{n}^{x}(s)=\sum_{n'}^{N-1}x_{n'}\psi *\left[\frac{(n'-n)\delta t}{s}\right]$$

where *n* is the localized time index, *s* is the wavelet scale, *δt* is the sampling period, *N* is the number of points in the time series, and the asterisk indicates the complex conjugate.

The wavelet coherency was used to identify frequency bands and time intervals where two times series were related [29]. The wavelet coherency *R*_*n*_ is defined as:

(2) $$R_{n}^{2}(s)=\frac{{\left|\langle s^{-1}w_{n}^{XY}(s)\rangle \right|}^{2}}{\langle s^{-1}{\left|w_{n}^{X}(s)\right|}^{2}\rangle \langle s^{-1}{\left|w_{n}^{Y}(s)\right|}^{2}\rangle }$$

where, 〈 〉 indicates smoothing in both time (using Gaussian function) and scale (using Boxcar filter). The statistical significance level of the wavelet coherence is estimated using Monte Carlo methods [29].

### 3.3. Mann-Kendall Test

Trends in annual time series of SM and T were estimated and tested at the 5% significance level using the non-parametric Mann-Kendall test applied to MRB from 1950 to 2010, which is robust and distribution independent. It is a nonparametric trend test that has been widely used in studies dealing with hydro-climatic analysis [1] [32]. For the trends of the SM and air T, the null and alternative hypothesis was used, with the no trend in the null and a significant trend in the alternative hypothesis. We not only estimated the trends for SM and T, but also intensively quantified the Man-Kendall cross relation value between SM and T on a grid by grid basis for over 18,000 grids in the MRB.

## 4. Results

### 4.1. QRM Results

**Figure 1.**
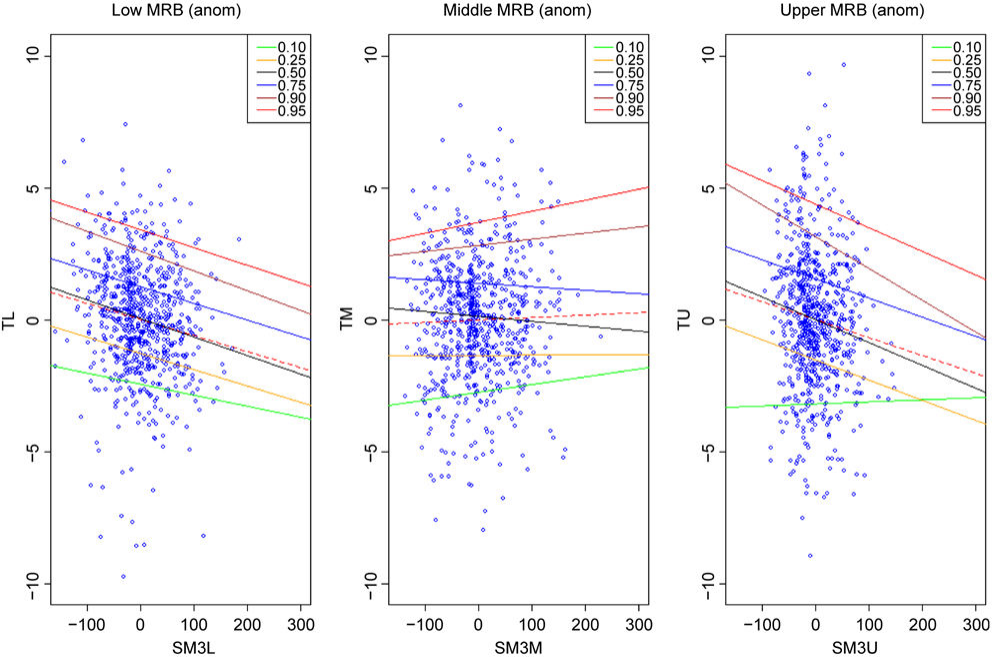
Relationships between monthly anomaly soil moisture and anomaly air temperature for the lower, middle, and upper MRB. The X-Axis represents the anomaly soil moisture (the 3rd layer) in the lower (SM3L), middle (SM3M), and upper (SM3U); the Y-Axis represents the anomaly air T in the lower (TL), middle (TM), and upper (TU). Solid lines denote regression for selected quantiles; dashed lines represent the means.

Figure 1 shows QRM scatter plots of monthly SM anomalies and monthly air T anomalies for the lower, middle, and upper MRB. Some interesting evidences which cannot be detected by mean regression are observed. The slopes of the regression lines are negative (drier soil results in higher T) for all the months and relationships are statistically significant in lower MRB. The slopes of the regression lines get steeper as the quantile increase from 10^th^ quantile till 90^th^ quantile, and the strongest negative slopes are associated with the 90^th^ quantile slope which is nearly twice those of 50^th^ quantile. The result is similar to the relation between SM and maximum air T in [7]. However, the most interesting result is that the slope of 90^th^ is slightly steeper than 95^th^, which indicates that increases in SM lead to greater declines in air T along the 90^th^ quantile than on the 95^th^ quantile (extreme hot weather). During extreme climate condition, increased air T with more evapotranspiration (ET), more moisture in atmosphere and then more precipitation, and SM does not typically contribute greatly to air T on average in 95^th^ quantile. Furthermore, the higher air T results in higher ET, which lowers the wetness of soil as well. The slopes for 50^th^ and 75^th^ quantiles are almost parallel, suggesting that air T in these quantiles did not vary dramatically with SM.

The most interesting QRM results happened in the middle MRB, the slopes of the regression lines are mixed with positive slopes (drier soil results in lower T) for lower quantiles (10^th^ and 25^th^ quantiles) and higher quantiles (90^th^ and 95^th^ quantiles), and negative slopes (drier soil results in higher T) for middle quan tiles (50^th^ and 75^th^ quantiles). The overall relations are more difficult to interpret because different areas of the domain experience different seasonal mixed precipitation (snow-domain transition to rain-domain) in different time periods. Slopes of 10^th^ and 95^th^ quantiles are somewhat steeper than for the 25^th^ and 90^th^ quantiles, respectively. These results are comparable to those shown in studies of [33] and are broadly consistent in their results on SM is most strongly associated with the high end of the temperature distribution. Slopes are positive in the areas of the domain with higher and lower temperatures. Slopes are negative for 50^th^ and 75^th^ quantile and broadly similar.

The upper MRB is strongly winter dominant, particularly at higher elevations. The winter temperature regimes largely determine how much of the winter P is typically stored as snow, and also play a strong role in determining the T sensitivity. The slope of 90^th^ quantile is steeper than that of 95^th^ quantile as that in the lower MRB. The highest slope happens in 90^th^ quantiles not 95^th^, which highlights the relation between SM and air T is weaker during extreme climate (95^th^ quantile). The slopes of the QRM quantiles are negative (drier soils result in higher T) for all the quantiles except for the 10^th^ quantile, which might due to the frozen soil during lower T in the upper MRB. These impressions are strengthened by considering a sequence of density plots based on the QRM estimates.

### 4.2. WTC Results

**Figure 2.**
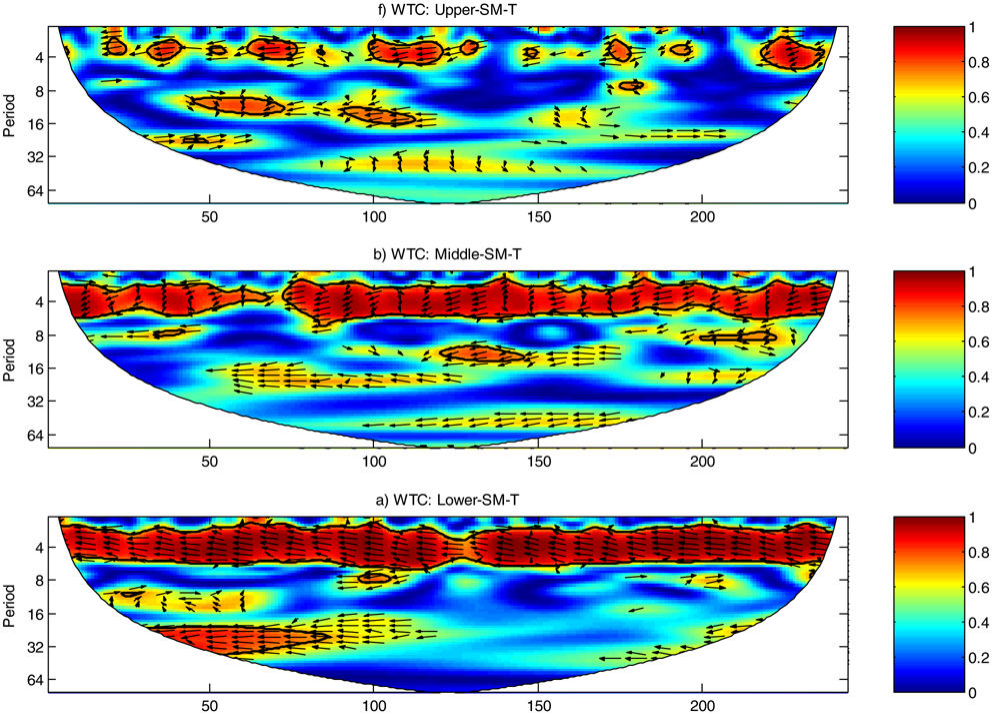
Wavelet coherence between the SM and T in the upper, middle, and lower MRB. The scales of the x-axis are seasons, and y-axis are months. The 95% significance level is shown as a thick contour. The thin solid line indicates the cone of influence. The relative phase relationship is shown as arrows (with in-phase pointing right, anti-phase pointing left). All significant sections show anti-phase behavior.

The relationship between SM and T are further examined by decomposing the time series of SM and T in time-frequency space in order to determine both the dominant modes of variability and how those modes vary in time (Figure 2) by computing the WTC. WTC was also used to identify the phase difference (lag time) between variables of SM and T during periods of significant coherence by plotting arrows. Arrows point right for time series in-phase and left for out-of-phase. Vertical arrows pointing up when SM leads T and down when T leads SM.

The WTC between SM and T in the upper, middle, and lower MRB were plotted in Figure 2. The contours represent statistically significant periods (95% significant), based on the Monte Carlo experiment. The most notable features are that SM and T are very well correlated at statistically significant level, with high coefficients in 1-yr period over the entire study period (1950–2010) for the middle and lower MRB, which means the two variables are strongly correlated on an interannual time scale. Consequently, regression techniques based on previous year’s SM and T can be used to predict the coming year T with considerable skill. The significant regions in 1-yr band are so extensive that it is very unlikely simply by chance. Arrows all point to left indicate a very distinct anti-phase relationship for a given wavelength between SM and T (e.g., drier soil is associated with higher air T). The average wavelet coherency between SM and T is greater than 0.85 and 0.9 for the 1-yr period for the middle and lower MRB, respectively. There is higher coherency in the 2 – 4 yr band in the middle MRB, which h is similar to the frequency of the El Niño Southern Oscillation (ENSO) (every 3 – 7 yr). The next best correlation band stands out being significant between SM and T is the 7 – 10 yr in the lower MRB, which indicates that the SM is strongly correlated to T at the similar frequency patterns as the Atlantic Multi-Decadal Oscillation (AMO) (every 7 – 15 yr). In addition to local climate impacts, large scale climate patterns/oceanic patterns also impact the land-surface interaction in MRB. Further investigation of the relationships between AMO, ENSO, and SM and T could be used in developing experimental air T forecasting applications.

Similar as the WTC for the middle and lower MRB, the WTC in the upper MRB also shows higher coherence in 1-yr band with rather lower coherency outside of this period throughout the study period in upper MRB. However, the coherency (averaged at 0.70) between SM and T in upper MRB in the 1-yr period is not as significant as it in the middle and lower MRB. These differences have to do with the air T that determine the precipitation regimes. The upper MRB is colder, the precipitation falls as snow and snow accumulation is the dominate driver of the overall SM. The sensitivity of air T to SM becomes relatively small and largely associated with snow water equivalent (SWE). The phase behavior out of 1-yr band for the SM and T does not show a strong coherency.

### 4.3. Mann-Kendall Test Results

For each 1/8^th^ degree grid cell, we quantified trends in monthly series of SM and T (not shown here) and tested at the 5% significance level using the non-parametric Mann-Kendall test applied to MRB from 1950 to 2010. The T trends, although shown to be generally positive (warmer) cross the entire MRB, are larger in the upper than in the middle and lower MRB. These trends are comparable to those shown in study of [4]. These T trends seem to be more strongly controlled by precipitation regimes at the regional scale. Contrary to the trends in T, the trends of SM are more mixed and difficult to analyze over the study period because SM has longer memory and not only controlled by short term climate variability in regions, but also by long-term processes like global warming and oceanic patterns. The trends of SM toward positive (wet) in upper and negative (dry) in lower domain, with no significant trends in part of middle MRB.

**Figure 3.**
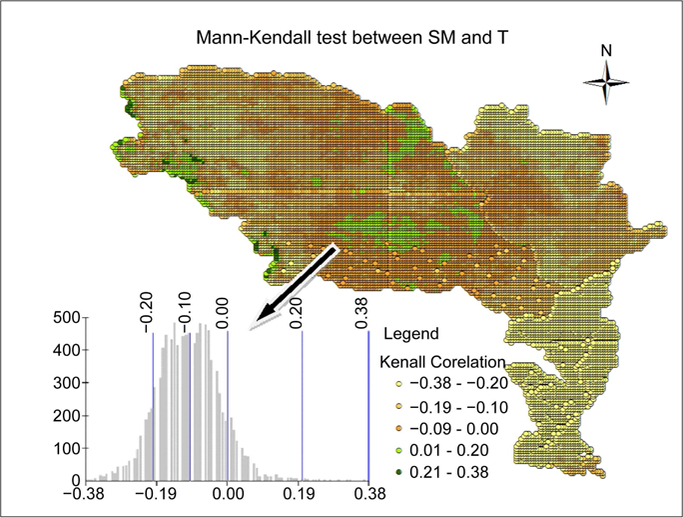
Mann-Kendall correlation coefficients between SM and T in the upper, middle, and lower MRB.

Using the Mann-Kendall test, we quantified the correlation between SM and T at annual scale for each grid (18,000 grids) over the study period and shown the results in Figure 3. Negative results between SM and T were pervasive the entire MRB, in simple terms, increasing in T (warmer condition) is associated with decreasing SM (drier condition). The negative relations have a larger expanse and magnitude in the lower MRB, this makes sense, because this part of domain get relatively less impacts of snowmelt on SM.

The highest negative relation is found at −0.38 in lower MRB, which is due to the warmer weather in lower domain compared to the upper area because SM has a strong impact on higher air T than lower air T [34]. The values of the negative relation mainly are relatively small in absolute value over much of the domain (in comparison with negative values), well they are generally negatively consistent, which is consistence with the results in QRM section. The grids with positive correlations are spotted in the upper and mainly in the middle MRB with statistically insignificant, which is also agreed with the results in QRM section (Section 4.1). SM only positively related to T in small region (7% of the MRB) of the study area.

## 5. Conclusions and Discussion

To our knowledge, no similar analysis was performed in the MRB, it is possible the first explicit investigation of the correlation between SM and T over an extended period (1950 to 2010) in the MRB. The results have significant implications for future water availability and temperature predictability. The major findings are:

1) QRM results show that SM negatively correlated to air T for the low and upper MRB, with the higher correlation at 90^th^ quantile. QRM slopes are mixed (negative and positive) for the middle MRB, because the middle MRB is the transient area with snow dominant to rain dominant.
2) The T and SM are strongly correlated in 1-yr period over the entire study period, indicating that the two variables are interannually correlated. The WTC correlations between SM and T in the 2 – 4 yr, 3 – 7 yr, and 7 – 10 yr bands are associated with ENSO and AMO, respectively.
3) The annual T and SM are significantly negatively related in the MRB from 1950 to 2010, indicating that the dry (wet) soil leads to warm (cool) weather in the MRB.

The QRM results indicated that the T is strongly correlate with SM in the study area, especially the high end of SM at 90^th^ quantile, however, the correlation decrease at the extreme climate condition (above 95^th^ quantile). The relationships between SM and T are also impacted by the regional climate which makes quartile slopes mixed in the climate transition zone, e.g., the middle of MRB.

To clearly demonstrate the relationship between SM and T, we muted the impacts of other variables (such as ET and P) by calculating the WTC between them. WTC between SM and T yielded values as high as 0.70, 0.85, and 0.9 in 1-yr band in upper, middle, and lower MRB, respectively, which indicate that a correlation between SM and T exists independent of other climate and hydrologic variability in the 1-yr band. In general, the WTC results are less convincing over the upper MRB than they are over the middle and lower MRB. Reasons for this may be due to impacts of snow on SM in the upper MRB.

We mapped grid cells with statistically significant Mann-Kendall relations between SM and T at annual scale in the MRB. It is likely the first time to quantify the value of the trends relation between SM and T in the MRB. One can infer from the map that the negative relation is the much more promising result. T is mostly negatively associated with SM. The T trends have been increased (warmer) over much of the domain during the study period.

The most likely and obvious application from the results is that the results provide insight for the land and atmosphere models fully coupling for climate change. Secondly, SM can sever as a potential predictor for T with a long land memory and strong land-atmosphere coupling, which is important for the future climate changes, especially global warming. Thirdly, the results help understanding the extreme climate because of SM’s drought index function.

Following this research, we have started land-atmosphere model coupling based on the relation between SM and T for historic, current, and climate change conditions.
